# Development and Optimization of CRISPR/Cas9-Assisted Recombineering in *Escherichia albertii*

**DOI:** 10.17912/micropub.biology.002076

**Published:** 2026-05-05

**Authors:** Shahab Ahmad Khan, Tara Marie Miller, Shantanu Bhatt, Edwin Li

**Affiliations:** 1 Department of Biology, Saint Joseph's University, Philadelphia, PA, US; 2 Saint Joseph's University, Philadelphia, PA, US

## Abstract

*Escherichia albertii* is a zoonotic pathogen frequently misidentified as diarrheagenic *E. coli* due to shared phenotypic and genetic traits, yet functional genomic studies in this species have been limited by the inefficiency of traditional genetic tools. To address this, we developed a bipartite CRISPR/Cas9-assisted lambda red (𝛌-Red) recombineering system for efficient, markerless genome editing in *E. albertii* . As proof of principle, we targeted the nonessential *lacZ* gene for negative selection by using Cas9 to generate lethal double stranded breaks in unedited cells. Using a recombinogenic single-stranded DNA (ssDNA) oligonucleotide to introduce a premature stop codon and an *NheI* restriction site, we achieved a recombination efficiency of 53%. Extending the induction time of the 𝛌-Red recombinase genes enhanced recombineering efficiency to 80%. This optimized CRISPR-assisted platform, the first reported application of its kind in *E. albertii,* enables the rapid generation of scarless mutations and provides a robust tool for the systematic analysis of *E. albertii* virulence factors and regulatory networks.

**Figure 1. CRISPR/Cas9-assisted lambda red recombination in E. albertii:** **(A)** A two-plasmid system was used to deliver the components necessary to create a double stranded break (DSB) in the *lacZ* gene. Following electroporation of *E. albertii* LMG20976 with pEcCas and induction of the lambda red genes, cells were transformed with pgRNA_Ealbertii, encoding an sgRNA complementary to the 5’ end of the *lacZ* ORF of *E. albertii* (underlined). A pgRNA expressing a noncomplementary sgRNA, pgRNA_Ecoli, was used as a control. For recombination, a recombinogenic ssDNA oligonucleotide with a premature stop codon and an *NheI* restriction site was used. Electroporation and recombination efficiencies were measured in cells after **(B)** 4-hr or **(C)** 24-hr induction of lambda red genes. Electroporation efficiency was measured as the total number of colonies forming units (CFU) per microgram of pgRNA_Ecoli or pgRNA_Ealbertii. Recombination efficiency was measured as the percentage of colonies with the *NheI* restriction site in cells co-electroporated with a recombinogenic ( *E. albertii* ) or nonrecombinogenic ( *E. coli* ) ssDNA oligonucleotide. A total of 36 colonies (12 colonies from each independent experiment) were tested for each co-electroporation experiment. Recombination efficiencies were not determined (ND) when cells were electroporated without a ssDNA oligonucleotide. **(D)** Colony PCR and *NheI* digestion were used to identify successful recombinants. PCR amplification with EL251106b and EL251106c primers yielded a 1,478 bp product (top gel). Successful oligonucleotide recombination generated products of 996 bp and 482 bp upon *NheI* digestion (bottom gel). Agarose gels from a representative 12 colonies from a co-electroporation experiment with pgRNA_Ealbertii and the recombinogenic ssDNA is shown. Lane 1: ladder; lane 2: wild type *E. albertii* colony; lanes 7, 9, 10: escapers; lanes 3, 5, 6, 8, 11-15: recombination-positive colonies. **(E)** Sanger sequencing using primer EL251106a confirmed that Cas9 targeted the *lacZ* segment in wild type *E. albertii* and lambda red recombinase introduced a stop codon and an *NheI* restriction site (boxes). **(F)** Blue-white screening on X-Gal plates confirmed the successful disruption of the *lacZ* gene. Representative colonies are shown for all the five different pgRNA electroporation experiments. Plate 1: pgRNA_Ecoli only, blue colonies; Plate 2: pgRNA_Ecoli + recombinogenic ssDNA, blue colonies; Plate 3: pgRNA_Ealbertii only, more than 90% white colonies; Plate 4: pgRNA_Ealbertii + nonrecombinogenic ssDNA, white and blue colonies; Plate 3: pgRNA_Ealbertii + recombinogenic ssDNA, more than 90% white colonies.

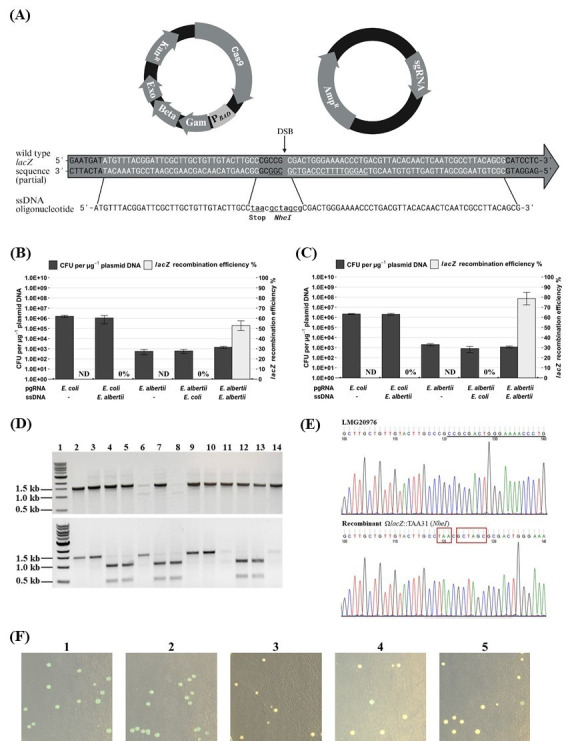



## Description

*Escherichia albertii* is a Gram-negative bacterium that was first identified as a distinct species in 2003, having been previously misclassified as *Hafnia alvei* (Albert et al., 1991; Bhatt et al., 2019). It is now recognized as a significant zoonotic pathogen with a host range that includes humans, poultry, wild birds, pigs, and other animal species. Despite its clinical importance, *E. albertii* is frequently underreported or misidentified as diarrheagenic *E. coli* because they share many phenotypic and genetic traits. Retrospective multilocus sequence typing (MLST) has revealed that approximately 15% of clinical isolates, that were implicated in prior outbreaks and originally classified as enteropathogenic *E. coli* (EPEC) or enterohemorrhagic *E. coli* (EHEC), were actually *E. albertii* , highlighting the persistent challenges in diagnostic accuracy (Ooka et al., 2015).

*E. albertii* , EPEC, and EHEC belong to the attaching and effacing (A/E) group of bacterial pathogens. A/E pathogens are defined by the locus of enterocyte effacement (LEE), a chromosomal pathogenicity island essential for the formation of A/E lesions on the intestinal epithelium (Posfai et al., 1997). Much of our knowledge on the genetic organization, regulation, and function of the LEE has been the result of studies conducted on EPEC and EHEC (Mellies et al., 2007; Wong et al, 2011; Franzin and Sircili, 2015; Bhatt et al., 2016). The LEE encodes a type III secretion system (T3SS) that directly links the cytoplasm of the bacterium with that of the host. Thereafter, the bacterium injects an assortment of effector proteins into the host cell that subvert signal transduction pathways to reorganize the host cytoskeleton that ultimately results in microvillar disintegration ( **effacement)** and intimate bacterial adherence ( **attachment** ) to the infected cell (Gomes et al., 2020). The LEE is indispensable for the virulence of A/E bacteria, which has prompted intense investigations into understanding the molecular mechanisms that regulate this morphogenetic element in EPEC and EHEC. Research has revealed that most environmental signals regulate the LEE via the three LEE-encoded transcriptional factors - Ler, GrlR, and GrlA (Platenkamp and Mellies, 2018).

Unlike EPEC and EHEC, the molecular pathogenesis of *E. albertii* remains largely a mystery, with studies restricted to comparative genomic analyses These studies revealed the conservation of the LEE, including the core transcriptional factors, Ler, GrlR, and GrlA, suggesting that the bacterium harbors the genetic capability to form A/E lesions with mechanistic similarity to EPEC and EHEC (Ooka et al., 2015, Egan et al., 2016). However, for many years, functional studies were restricted in *E. albertii* to extrapolation based on work conducted on other A/E bacteria. This was largely due to the absence of a reliable technique for targeted mutagenesis in the bacterium. Thus, even though its potential as an emerging pathogen has been globally recognized since its initial isolation almost 35 years ago, the molecular mechanisms and environmental signals that coordinate its virulence program have yet to be fully elucidated. Furthermore, the discovery of some strains harboring potent *stx _2a_* subtype Shiga toxin genes significantly enhances the virulence potential of *E. albertii* , yet the regulatory mechanisms governing such traits are not fully understood (Ooka et al., 2015). Consequently, there is a critical need to develop a functional genomics platform that enables the exploration of gene function in *E. albertii* and lays the foundation for further research.

A significant breakthrough was achieved with the development of a modified lambda red (𝛌-Red) recombineering protocol, which enabled the first site-specific deletions of both LEE-encoded ( *ler* , *grlRA* ) and non-LEE-encoded genes ( *hfq* ) (Egan et al., 2016). The ability to edit genes enabled researchers to test and experimentally validate longstanding predictions on the roles of these regulatory factors in modulating gene expression from the LEE and the resulting virulence of *E. albertii.* Despite the success, lambda red recombineering in *E. albertii* is technically inefficient, locus dependent, with restrictive output, in comparison to *E. coli* (Egan et al., 2016). These features limit the current use and scalability of the technique. Recent studies in other enteric bacteria have highlighted the utility and scalability of using CRISPR/Cas9 and lambda red synergistically with high recombination efficiencies (Pyne et al, 2016; Su et al., 2020; Tahir et al., 2022). However, the use of CRISPR/Cas9 technology has not yet been explored in *E. albertii* .

The integration of CRISPR/Cas9 with the lambda red system has revolutionized genome editing in other members of the *Enterobacteriaceae* . Initially adapted for *Escherichia coli* and CRISPR *Streptococcus pneumoniae* (Jiang et al., 2013), this combinatorial approach overcomes the primary limitation of lambda red alone—its reliance on selectable markers and the subsequent "scars" left behind after marker excision. In this bipartite system, lambda red facilitates the homologous recombination of donor DNA, while CRISPR/Cas9 serves as a programmable negative selection tool that induces lethal double-strand breaks in unedited cells, effectively killing the wild-type population (Pyne et al., 2015; Reisch and Prather 2015). This coupling facilitates the rapid generation of markerless, scarless mutations with near 100% efficiency. For example, in *E. coli* , CRISPR-assisted systems have been successfully used to perform large-scale chromosomal deletions of up to 19.4 kb and the insertion of up to 3 kb of heterologous DNA without the need for extensive screening (Pyne et al., 2015). 𝛌-Red-assisted recombination with CRISPR/Cas9 has recently been used to integrate a 12 kb lycopene biosynthetic pathway into the *E. coli* chromosome in a single step (Su et al., 2020). Furthermore, this approach has been applied to target multiple virulence factors, such as the knockout of quorum-sensing ( *luxS* ) and adhesion genes ( *fimH* , *bolA* ), leading to significant reduction in biofilm formation and providing a more robust framework for systematic functional genomics over traditional recombineering methods alone (Alshammari et al., 2023).

To demonstrate chromosomal gene editing in the *E. albertii* strain LMG20976 with CRISPR/Cas9-assisted recombineering, the nonessential *lacZ* gene was targeted using a two-plasmid based system ( Fig. 1A ). The plasmid pEcCas coexpresses Cas9 and lambda red recombinase under the control of the native *cas9* promoter and the arabinose-inducible *P _BAD_* promoter (Li et al., 2020), respectively, whereas derivatives of the plasmid pgRNA-bacteria express the single guide RNA (sgRNA) of interest from the minimal synthetic promoter J231119 (Lei et al., 2013). To direct the Cas9-dependent double stranded DNA cleavage, an sgRNA complementary to the 5’ end of the *lacZ* ORF of *E. albertii* was expressed from the plasmid pgRNA_Ealbertii. The control plasmid used, pgRNA_Ecoli, expresses an sgRNA complementary to the *lacZ* gene from *E. coli* but not *E. albertii.* This sgRNA is not expected to introduce a dsDNA break in the native *lacZ* gene of *E. albertii* but would serve to establish a baseline for transformation and recombination without Cas9 selection. A recombinogenic single stranded oligonucleotide was designed to incorporate a premature stop codon and an *NheI* restriction site for verification of recombinants. By contrast, a nonrecombinogenic ssDNA oligonucleotide with homology to the *E. coli lacZ* gene was used as a negative control ( Fig. 1A ).

Following a 4-h induction of the 𝛌-Red recombinase, *E. albertii* cells were electroporated with the plasmid pgRNA_Ecoli, either alone or in combination with the recombinogenic ssDNA oligonucleotide, yielding transformation efficiencies of ~1 x 10 ^6^ CFU/µg of plasmid DNA. In contrast, electroporation of *E. albertii* with the plasmid pgRNA_Ealbertii, either alone or in combination with the nonrecombinogenic ( *E. coli* ) or recombinogenic ( *E. albertii* ) oligonucleotide, resulted in a comparable 3-log reduction in transformation efficiency ( Fig. 1B ). This significant decrease in cell viability is attributed to lethal CRISPR/Cas9-mediated cleavage of the unmodified *lacZ* locus. A small fraction of pgRNA_Ealbertii transformants survived Cas9-mediated cleavage in the absence of the ssDNA template (<0.1% relative to the pgRNA_Ecoli). These escapers, also observed to a lesser extent in the presence of the ssDNA template, likely arose from spontaneous mutations within the targeting spacer of the gRNA or from loss-of-function mutations affecting the expression or activity of Cas9. Furthermore, some escapers possibly evaded death through random recombination mediated by the 𝛌-Red system or through the alternative-end joining DNA repair mechanism (Murphy and Campellone 2003; Chayot et al., 2010; Jiang et al., 2013; Cui and Bikard, 2016; Li et al., 2023). Notably, transformation efficiencies were comparable after extending the induction period to 24 h ( Fig. 1C ).

A subset of the observed transformants were genotyped using colony PCR followed by *NheI* digestion to identify successful edits ( Fig. 1D ). Across three independent experiments, screening a total of 36 colonies yielded a recombination efficiency of 53%, after inducing the lambda red genes for 4 hours ( Fig. 1B ). Extending the induction time to 24 hours further enhanced gene editing, yielding a 1.5-fold increase in recombination efficiency to ~80% ( Fig. 1C ). As expected, the *NheI* recognition site was absent in all 36 colonies screened from the control groups. Successful incorporation of both the premature stop codon and the *NheI* sequence was further confirmed by DNA sequencing ( Fig. 1E ). Additionally, blue-white screening identified transformants harboring either intended (recombinants) or random (escapers) *lacZ* mutations that appeared as white colonies ( Fig. 1F, plates 3-5). Taken together, these results demonstrate the adaptability of the CRISPR/Cas-assisted recombineering as an effective tool for genome editing in *E. albertii* .

To the best of our knowledge, this study provides the first experimental demonstration of the CRISPR/Cas9-lambda red bipartite system for targeted gene editing in *E. albertii.* Although recombineering-based approaches for gene editing have been used before in *E. albertii* , the reported recombination frequencies were exceedingly low, resulting in limited gene editing and mutant engineering (Egan et al., 2016). By contrast, the CRISPR/Cas9-lambda red bipartite system described here overcomes this technical bottleneck, which will enable researchers to modify previously unalterable genetic loci. Another advantage of our system is that it facilitates markerless gene edits, thus enabling the construction of mutants without the reliance on selectable markers.

## Methods

**Bacterial strains, plasmids, primers, oligonucleotides, and growth conditions**

Bacterial strains, plasmids, and oligonucleotides used in this work are listed in Tables 1 and 2. *E. coli* DH5α (New England Biolabs) was used to maintain and propagate the plasmid pEcCas (Addgene #73227), pgRNA-bacteria (Addgene #44251) and its derivatives. pEcCas carries the *cas9* , lambda red, and kanamycin resistance genes. The pgRNA-bacteria backbone was used to synthesize (Genscript) pgRNA_Ealbertii, encoding a specific sgRNA targeting the *lacZ* gene in *E. albertii* ; conversely, a non-targeting control pgRNA was constructed using a spacer sequence corresponding to the *E. coli lacZ* gene (pgRNA_Ecoli). All the oligonucleotides were synthesized by Integrated DNA Technologies (IDT) at the 25-nm scale using standard desalting.

Bacterial strains were first streaked onto Luria-Bertani (LB) agar plates supplemented with or without antibiotics and then grown aerobically in LB broth culture tubes at 37°C and 180 rpm for ~16-20 hours. The recombinant strains carrying plasmids pEcCas and pgRNA were selected on kanamycin and ampicillin at concentrations of 50 μg/mL and 100 μg/mL, respectively.

**Table:** 

**TABLE 1** Strains and plasmids used in this study		
**Strains**	**Relevant Characteristics **	**Source or Reference **
DH5α	*E. coli* F− *endA1 glnV44 thi-1 recA1 relA1 gyrA96 deoR nupG* ϕ80d *lacZ* ΔM15 Δ( *lacZYA-argF* ) *U169* , *hsdR17* (rK− mK+) λ−	New England Biolabs #C2988J
LMG20976	*E. albertii* wild-type strain	Bhatt’s lab
EL1001	LMG20976 transformed with pEcCas, Km ^R^	This study
EL1101	EL1001 transformed with pgRNA_Ealbertii, Km ^R^ Ap ^R^	This study
EL1102	EL1101 Ω *lacZ* ::TAA31 ( *NheI* )	This study
**Plasmids**		
pEcCas	Bacterial expression vector containing the *S. pyogenes* *cas9* and Red recombinase genes ( *cas9; gam; exo; beta* )	Addgene ID #73227
pgRNA-bacteria	Bacterial vector for the expression of a customizable guide RNA (gRNA)	Addgene ID #44251
pgRNA_ *Ecoli* (control)	Single guide RNA targeting the *E. coli* *lacZ* gene (spacer sequence: TCGCACAGCGTGTACCACAGCGG)	This study
pgRNA_ *Ealbertii*	Single guide RNA targeting the *E. albertii* *lacZ* gene (spacer sequence: CAGGGTTTTCCCAGTCGCGG)	This study

**Table:** 

**TABLE 2** Oligonucleotides used in this study		
**Oligonucleotides **	**Sequences **	
ssDNA ( *E. albertii* )	ATGTTTACGGATTCGCTTGCTGTTGTACTTGCCTAACGCTAGCGCGACTGGGAAAACCCTGACGTTACACAACTCAATCGCCTTACAGCG	
ssDNA ( *E. coli* )	CTTTAACGCCGTGCGCTGTTCGCATTATCCGAACCATCCGTAGATAGGCTAGCCTGTGGTACACGCTGTGCGACCGCTACGGCCTGTATG	
**Primers**		
EL251106b forward/5’ primer	GGCGAACATATTGCGTAC	
EL251106c reverse/3’ primer	CGCTCAGGTCAAAGTCAG	
EL251106a sequencing forward/5’ primer	CGTTAGAGCGATAAGC	

**Plasmid DNA isolation and purification**

Plasmid purification and cleanup were performed using commercial spin column kits from Zymo Research according to the manufacturer's instructions. 

**Preparation of recombinogenic DNA**

The ssDNA oligonucleotides for recombineering experiments, a recombinogenic ssDNA oligonucleotide ( *E. albertii* ) and a nonrecombinogenic ssDNA (control, *E. coli* ), were designed through SnapGene and A plasmid Editor (ApE). An inframe stop codon (TAA) followed by an *NheI* restriction site was inserted into the *lacZ* open reading frame to facilitate the screening of successful recombinants.

**Preparation of electrocompetent cells**

*E. albertii* LMG20976 wild-type strain was streaked on LB agar supplemented with tetracycline (15 µg/mL). A single colony was inoculated into 5 ml of LB and incubated overnight at 37°C under shaking (180 rpm) conditions . The following day, the culture was diluted 1:100 into 40 mL LB medium, and grown under the same conditions to an OD _600_ of 0.4-0.6. Cells were then washed four times with ice-cold 10% glycerol and resuspended in 530 μL of ice-cold 10% glycerol. Approximately 90 μL of electrocompetent cells were aliquoted into microcentrifuge tubes. Electroporation was performed at a voltage of ~1.8 kV. After electroporation the cells were recovered in 1 mL of LB for 2 hr at 37°C (180 rpm).

**CRISPR/Cas9-assisted lambda red recombineering**

Recombineering was performed using a two-step sequential electroporation protocol. In the first electroporation step, wild-type *E. albertii* LMG20976 was transformed with 400 ng of pEcCas. Resulting transformants were then prepared for a second electroporation to introduce pgRNA (pgRNA_Ealbertii or pgRNA_Ecoli) with or without a ssDNA template. Two induction strategies were used to induce the lambda red genes on pEcCas during the preparation of electrocompetent cells: 1) a long induction (~24 h) in which cells were cultured overnight (18-20 h) at 37°C (180 rpm) in LB broth supplemented with 10 mM arabinose, subcultured (1:50) the next day into 40 mL LB containing 10 mM arabinose, and grown to an OD _600_ of 0.6- 0.7 (~4 h); and 2) a short induction (~4 h) in which arabinose was present only during the subculture growth phase. Following the induction, cells were made electrocompetent as described above.

For recombineering, using ssDNA oligonucleotide, the second electroporation step consisted of 250 ng of targeting pgRNA_Ealbertii or non-targeting pgRNA_Ecoli, mixed with approximately 2 μg of recombinogenic ( *E. albertii* ) or non-recombinogenic oligonucleotide ( *E. coli* ). Following recovery, cell cultures were diluted, and 0.1-150 µL equivalents were plated onto selective LB agar plates containing kanamycin (50 µg/mL), ampicillin (100 µg/mL) , isopropyl β-D-1-thiogalactopyranoside (IPTG) (100 mM), and 5-bromo-4-chloro-3-indolyl-β-d-galactopyranoside (X-Gal) (10 mM). Electroporation efficiency was determined by counting the total number of colonies forming units (CFU) generated per μg of pgRNA plasmid.

**Recombinant screening and verification**

Recombination of *lacZ* ssDNA oligonucleotides was screened by colony PCR followed by *NheI* digestion. Briefly, a 1,478 bp fragment of *lacZ* was amplified by colony PCR using the primers EL251106b and EL251106c with the Taq 5X Master Mix (New England Biolabs). The amplified PCR product was restricted with *NheI* to screen for successful recombinants. The integration of the ssDNA oligonucleotide introduces an *NheI* restriction site; consequently, successful recombinants were identified by the cleavage of the PCR product into two distinct fragments of 996 bp and 482 bp. Recombination efficiency was measured by enumerating the number of correctly edited colonies from the pool of total viable colonies electroporated with either the recombinogenic ssDNA ( *E. albertii* ) or nonrecombinogenic ssDNA ( *E. coli* ). The PCR product was sequenced by Sanger sequencing (Genewiz) using the primer EL251106a to confirm the precision of the CRISPR-assisted recombineering and the absence of unintended mutations at the target site.
