# Erratum: Development and Optimization of CRISPR/Cas9-Assisted Recombineering in *Escherichia albertii*

**DOI:** 10.17912/micropub.biology.002214

**Published:** 2026-05-26

## Description

Erratum: Khan SA, Miller TM, Bhatt S, Li E. 2026. Development and Optimization of CRISPR/Cas9-Assisted Recombineering in *Escherichia albertii* . microPublication Biology. 10.17912/micropub.biology.002076.

In the version of this microPublication originally published online, the last sentence of the figure legend incorrectly referred to "Plate 3" when the correct plate is Plate 5. This error has been corrected in the online version.

